# Integrated drought monitoring and analysis: A novel framework based on multi-source remote sensing data and ensemble machine learning

**DOI:** 10.1371/journal.pone.0346060

**Published:** 2026-04-21

**Authors:** Pengchao Dong, Dexiang Gao, Tao Wen

**Affiliations:** 1 School of Architectural Engineering, Zhengzhou University of Industrial Technology, Zhengzhou, China; 2 School of Atmospheric Sciences, Sun Yat-sen University, Southern Marine Science and Engineering Guangdong Laboratory (Zhuhai), Zhuhai, China; 3 School of Geosciences, Hubei Key Laboratory of Complex Shale Oil and Gas Geology and Development in Southern China, Yangtze University, Wuhan, China; University of Nevada Las Vegas, UNITED STATES OF AMERICA

## Abstract

Against the backdrop of climate change, drought risks are escalating in critical agricultural regions, highlighting the need for effective monitoring tools. Existing in-situ and remote sensing-based drought monitoring methods suffer from low accuracy, poor spatial representativeness, and insufficient explanatory power. To address these gaps, we propose a novel framework integrating multi-source remote sensing data and an ensemble machine learning (ML) model. This approach was validated using the Beijing-Tianjin-Hebei-Shandong-Henan region in China as a case study. The results of this study indicate that the Bayesian-weighted ensemble model effectively captures the nonlinear relationships between drought and its driving factors across multiple time scales (1, 3, 6, and 12 months), thereby enhancing prediction accuracy. For the Standardized Precipitation Evapotranspiration Index (SPEI), the model achieves R^2^ values ranging from 0.71 to 0.74 across the four time scales. Additionally, it attains over 78% accuracy in classifying different drought severity classes, with a 98% accuracy rate for extreme drought detection. Correlation analysis identifies precipitation anomalies (Pa, R = 0.31) and potential evapotranspiration (PET) as key correlates of short-term drought (SPEI-1). SHAP (SHapley Additive exPlanations) further quantifies their contribution at 21% each, confirming them as dominant drivers. For long-term drought, correlation analysis shows soil moisture is critical (R > 0.27, P < 0.001), SHAP ranked Palmer Drought Severity Index (PDSI) among the strongest predictive features, while soil moisture remained an important physically interpretable driver. The model successfully captured the severe drought event in June 2019 within the study area and elucidated the spatiotemporal evolution characteristics of droughts across different time scales. This study provides a novel, effective tool for regional drought monitoring and analysis, enhances the interpretability of drought drivers through SHAP analysis, and offers a scalable framework to support data-driven drought risk management across agricultural regions.

## 1. Introduction

Drought, a pervasive and complex natural disaster induced by climatic variability, poses significant challenges to agricultural sustainability, water resource management, and socioeconomic stability in arid and semi-arid regions [1]. The Beijing–Tianjin–Hebei–Shandong–Henan region is a critical agricultural zone in China with a temperate monsoon climate and highly uneven precipitation, making it especially vulnerable to recurrent droughts [2]. Traditional station-based drought monitoring approaches suffer from poor spatial continuity and fail to capture synergistic interactions among precipitation deficits, vegetation stress, soil moisture depletion, and temperature anomalies [3]. While remote sensing technologies have advanced drought characterization by providing spatially explicit data (e.g., NDVI/EVI for vegetation health, LST, precipitation), conventional remote sensing-based models often rely on single-index analyses, neglecting the multifaceted drivers of drought dynamics [4–7].

Recent advances in machine learning (ML) and deep learning (DL) have shown potential in addressing these limitations via multi-source data integration. For instance, Shen et al. (2019) developed a deep feedforward neural network (DFNN) to fuse MODIS and TRMM data for drought assessment, achieving robust correlations with soil moisture and standardized indices. Similarly, Zhou et al. [8] (2024) proposed an attention-weighted LSTM (AW-LSTM) model for drought monitoring in the Huang-Huai-Hai region, highlighting temporal feature extraction utility.

Existing ML/DL drought models face key gaps: poor capture of spatiotemporal dependencies in heterogeneous regions and lack of interpretability for drought driver contributions [9–11]. While prior ensemble ML studies show promise [12,13], most rely on arbitrary weighting (e.g., equal weighting) rather than data-driven optimization, limiting adaptability to diverse climatic/topographical regions like the Beijing-Tianjin-Hebei-Shandong-Henan area [14–17].

Effective integration of multi-source data with advanced ML approaches still faces several limitations. Such integration should simultaneously address spatial feature extraction, temporal dependency modeling, and interpretability. Current studies predominantly focus on model accuracy but overlook the mechanistic understanding of drought drivers, limiting their utility for adaptive resource management [18,19]. Additionally, conventional ML models (e.g., Random Forest, support vector machine) often underperform in handling high-dimensional data and non-linear relationships compared to DL architectures, yet their comparative efficacy in drought prediction remains inadequately assessed [20,21]. The lack of explainability in ML models highlights the need for frameworks that quantify the influence of drivers on drought outcomes. SHAP (SHapley Additive exPlanations) analysis offers a robust approach to effectively quantify and interpret the contribution of each variable, providing clear insights into the role of each driver. The combination of ML and SHAP provides a new approach for analyzing the drivers of drought.

To address the limitations of existing drought monitoring methods, this study introduces a novel Bayesian-optimized ensemble machine learning (ML) framework that integrates multi-source remote sensing data, such as MODIS and TerraClimate, for multi-scale drought prediction in the Beijing-Tianjin-Hebei-Shandong-Henan region. The framework begins with the analysis of spatiotemporal drought patterns from 2000 to 2020 to identify key drivers of drought across seasonal and interannual scales. It then dynamically combines predictions from Random Forest (RF), Extreme Gradient Boosting (XGBoost), Support Vector Regression (SVR), and Deep Feedforward Neural Networks (DFNN), with Bayesian optimization employed to **o**ptimize ensemble weights by maximizing validation performance. To assess the performance of this framework, comparative evaluations are conducted against benchmark models (RF, XGBoost, SVR, DFNN) using multi-scale Standardized Precipitation Evapotranspiration Index (SPEI) indices (SPEI-1, SPEI-3, SPEI-6, SPEI-12). Furthermore, SHAP analysis is utilized to quantify the contributions of key drought drivers, including precipitation anomalies, evapotranspiration, and vegetation stress, enhancing model transparency and interpretability. This study proposes a BOA-optimized weighted ensemble for multi-timescale SPEI prediction and drought severity classification within a unified framework. The key novelty is the integration of data-driven ensemble weighting with SHAP-based, scale-aware interpretability, enabling consistent identification of dominant drought drivers across timescales. The proposed framework improves the accuracy and robustness of drought monitoring and prediction, providing a scalable tool to support drought risk management in agricultural regions.

## 2. Materials

### 2.1. Case study area

**Fig 1:**
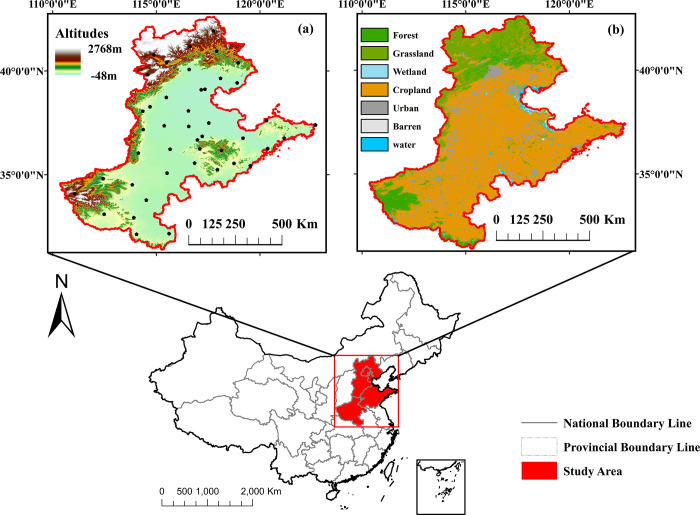
The geographical location map of the study area (a) displays the elevation distribution and meteorological station locations in the Beijing-Tianjin-Hebei-Shandong-Henan region, while the land cover map (b) categorizes all land types into seven classes: forest, grassland, wetland, cropland, urban, barren, and water.

The Beijing-Tianjin-Hebei-Shandong-Henan Region (32°–42°N, 111°–122°E), spanning approximately 530,000 km^2^, is a vast alluvial plain in eastern China. Elevations range from −15–1,406 m, predominantly featuring flat lowlands interspersed with sporadic low hills. The geographical location of the study area is shown in Fig 1, with subfigures (a) and (b) depicting detailed information on the elevation and land cover of the study area, respectively. Climatologically, the region experiences a temperate monsoon climate, with annual temperatures of 8–15 °C and precipitation of 480–1,050 mm, characterized by pronounced north-south precipitation gradients and significant interannual variability, leading to recurrent droughts and floods [22,23]. The Yellow and Huaihe Rivers form the primary hydrological system, sustaining the region’s 214,000 km^2^ of arable land. Corn cultivation spans 60,000 km^2^, making it a vital component of the regional agricultural economy. However, recurrent droughts pose significant challenges to yield stability and food security.

### 2.2. Data

In this study, drought indices were analyzed by leveraging remote sensing data as input parameters for an ML model. A detailed description of the datasets is shown in Table 1. Precipitation (CHIRPS), temperature (ERA5), and soil moisture (TerraClimate) were sourced from established repositories. Vegetation and thermal variables derived from MODIS include leaf area index (LAI), NDVI, evapotranspiration (ET), and LST. NDVI and ET data underwent quality control to remove invalid pixels based on the corresponding product quality-control documentation. Original temporal resolutions (daily to 16-day) were transformed into monthly means without weighting. All datasets were resampled to a 1,000 m resolution using nearest‐neighbor interpolation to ensure spatial consistency, and all spatial data were projected to the WGS84 coordinate system.

**Table 1:** Data sources and specifications of remote sensing variables used for drought monitoring.

Data Sources	Product	Variables (units)	Temporal Resolution	Spatial Resolution
UCSB-CHG	CHIRPS	Precipitation (mm)	Monthly	${\text{0}.\text{05}}^{\circ }\times {\text{0}.\text{05}}^{\circ }$
ECMWF	ERA5	Temperature (°C)	Monthly	${\text{0}.\text{1}}^{\circ }\times {\text{0}.\text{1}}^{\circ }$
WorldClim	Terraclimate	Soil moisture (mm)	Monthly	~4638.3 m
MODIS	MOD15A2H	LAI	8 days	500 m
	MOD13A1	NDVI	16 days	500 m
	MOD16A2	ET (mm)	8 days	500 m
	MOD11A1	LST (°C)	daily	1000 m

#### 2.2.1. MODIS data.

The MODIS, a multispectral sensor aboard NASA’s Terra and Aqua satellites, delivers global-scale biophysical datasets critical for environmental monitoring. This study utilized four MODIS products: MOD13A1 (16-day composite, 500 m, NDVI), MOD11A1 (daily composite, 1 km, LST), MOD16A2 (8-day composite, 500 m, ET), and MOD15A2H (8-day composite, 500 m, LAI). These datasets were sourced from NASA’s Level-1 and Atmosphere Archive & Distribution System (LAADS) (https://ladsweb.modaps.eosdis.nasa.gov/, accessed 27 January 2025). The NDVI and LST data were further processed to derive the Vegetation Condition Index (VCI) and Temperature Condition Index (TCI), serving as key inputs for drought assessment models. MODIS’s strengths lie in its harmonized global coverage, continuous data continuity since 2000, and synergistic multi-parameter outputs (e.g., simultaneous vegetation and thermal metrics), which collectively augment the robustness of large-scale hydrological analyses.

#### 2.2.2. CHIRPS and ERA5 data.

Precipitation (mm) data were sourced from the Climate Hazards Group Infrared Precipitation with Stations (CHIRPS), a high-resolution (0.05°) hybrid dataset integrating satellite estimates and ground observations (2000–2020), chosen for its enhanced accuracy in capturing spatial rainfall variability compared to ERA5 reanalysis. CHIRPS data were utilized to calculate both the Precipitation Condition Index (PCI) [24] and the Pa, representing monthly deviations from long-term averages. Temperature data were acquired from ERA5-Land’s 2-meter air temperature (temperature_2m), a 0.1° resolution reanalysis product interpolated to surface level with atmospheric stability adjustments. All datasets were accessed via Google Earth Engine (GEE) (https://developers.google.com/, accessed 27 January 2025), preprocessed to monthly temporal resolution, and spatially aligned for drought model integration.

#### 2.2.3. TerraClimate data.

TerraClimate is a high-resolution (~4 km) global dataset providing monthly climate variables (1958–2020), including precipitation (mm), temperature (°C), and water balance parameters. A dedicated water balance model integrates evapotranspiration, precipitation, and soil water capacity to generate variables like soil moisture. In this study, TerraClimate’s soil moisture data were used to calculate the Soil Moisture Condition Index (SMCI), while the Palmer Drought Severity Index (PDSI), a standardized metric integrating precipitation and temperature to quantify long-term hydrological anomalies, complemented drought analysis. PDSI values classify drought severity from extreme wetness (−4) to extreme drought (+4). Data were accessed via the University of Idaho’s public repository (http://www.climatologylab.org/terraclimate.html, accessed 27 January 2025).

#### 2.2.4. Meteorological station data.

Daily precipitation and temperature data were obtained from the National Oceanic and Atmospheric Administration (NOAA) (https://www.ncei.noaa.gov/data/, accessed 27 January 2025). Stations with missing records exceeding three consecutive months were excluded. Daily values were aggregated into monthly averages (temperature) and cumulative sums (precipitation). The SPEI at 1-, 3-, 6-, and 12-month scales (SPEI-1, SPEI-3, SPEI-6 and SPEI-12) was calculated following Vicente-Serrano et al.’s climatic water balance framework [25]. Negative SPEI values indicate moisture deficits, with magnitude corresponding to drought severity. Spatial analysis focused on 30 stations within the Beijing-Tianjin-Hebei-Shandong-Henan region (Fig 1), ensuring geographical consistency with the study area. Data homogeneity was verified via NOAA’s quality control flags, and snow-influenced periods were excluded to better align with the region’s hydroclimatic regime.

## 3. Methodology

### 3.1. Machine learning models

This study develops a Bayesian-optimized ensemble framework integrating four ML algorithms: RF, XGBoost, SVR, and DFNN. These models were selected to maximize learner diversity while maintaining strong predictive capability for nonlinear drought-related processes. Each algorithm contributes unique strengths: RF captures nonlinear relationships through ensemble decision trees, XGBoost minimizes prediction errors via gradient boosting, SVR handles high-dimensional data with kernel tricks, and DFNN identifies hierarchical patterns via multilayer perceptrons. These models differ in how they fit nonlinearities, interactions, and error structures; therefore, they are expected to produce partially complementary predictions rather than redundant outputs. The BOA-based ensemble weighting strategy was therefore adopted to combine these heterogeneous learners in a data-driven manner, so that the final prediction can benefit from their complementary strengths while reducing the risk of over-reliance on any single model. Details of the hyperparameter search space and the optimal parameter settings for each algorithm are summarized in S1 Table in the Supporting Information.

#### 3.1.1. Random Forest (RF).

RF is an ensemble algorithm that combines multiple decorrelated decision trees to enhance prediction stability and accuracy [26]. It operates by training each tree on a bootstrap sample of the dataset and splitting nodes using random subsets of features, thereby reducing overfitting through aggregated predictions. The final output is determined by majority voting (classification) or averaging (regression). RF inherently evaluates feature importance by measuring the impact of variables on prediction accuracy across all trees. Its robustness in handling noisy or high-dimensional data, such as multi-source drought indicators, makes it a widely adopted tool for complex environmental modeling tasks.

#### 3.1.2. Extreme Gradient Boosting (XGBoost).

XGBoost is a gradient-boosting framework that iteratively constructs decision trees to minimize residual errors from preceding models [27]. By incorporating regularization techniques, it penalizes model complexity to prevent overfitting while enhancing computational efficiency. The algorithm optimizes loss functions using gradient descent and supports parallel processing for scalable training. XGBoost excels in capturing intricate nonlinear relationships, such as those between meteorological variables and drought indices, and efficiently handles missing data through sparsity-aware splitting strategies. Its flexibility and high prediction accuracy make it particularly effective for drought scenarios requiring precise temporal and spatial resolution.

#### 3.1.3. Support Vector Regression (SVR).

SVR employs kernel functions to map input data into high-dimensional space, enabling linear regression on nonlinear patterns [28]. By constructing an epsilon-insensitive tube around the predicted values, SVR minimizes structural risk while tolerating minor deviations. Kernel selection (e.g., radial basis function) critically determines its ability to model complex interactions. Although computationally intensive for large datasets, SVR’s resistance to overfitting and effectiveness with small samples make it valuable for drought analysis in data-scarce regions.

#### 3.1.4. Deep Feedforward Neural Network (DFNN).

DFNN is a multi-layer feed-forward neural network characterized by the forward propagation of signals through successive layers. The model typically consists of an input layer, multiple hidden layers, and an output layer, with depth provided by the hierarchical hidden layers. In this study, the architecture design and parameter configuration of the DFNN are implemented using the “Keras” Python library.

### 3.2. Mann-Kendall trend analysis

The Mann-Kendall (M-K) [29] test was employed to detect trends and identify abrupt change points in the hydrometeorological time series data. We applied this method to analyze rainfall trends.. Results from the M-K analysis, including the Z_mk statistic and the intersection of UF/UB sequences, informed our understanding of long-term drought patterns and their potential drivers in the study region. Compared to parametric approaches, the M-K test requires no predefined functional forms, rendering it particularly suitable for analyzing long-term, complex datasets in climate and environmental studies [30]. In drought research, it effectively uncovers evolving patterns of multi-scale drought indices, such as via UF-UB curves to analyze abrupt shifts in precipitation or soil moisture sequences. Its algorithmic simplicity and robustness have established it as a cornerstone tool for climate diagnostics and forecasting.

### 3.3. Bayesian optimization algorithm (BOA) for Ensemble Weighting

The BOA [31] is an evolutionary strategy that refines solutions by leveraging probabilistic modeling through Bayesian networks. It begins by initializing a population of solutions uniformly across the search space. Iteratively, it evaluates candidate solutions using a fitness function, selects high-performing individuals, and constructs a Bayesian network to model their interdependencies. New solutions are then generated by sampling this network, ensuring preservation of learned variable interactions, and integrated into the population to replace less optimal candidates.

A key role of the Bayesian optimization approach employed in this study is to determine the optimal combination of ensemble weights by maximizing predictive performance on a validation set. For an input vector x, each base learner produces a prediction $y_{k}(x)$, where k ∈{RF,XGB,SVR,DFNN}. The ensemble prediction is formulated as a weighted average of individual model outputs:


$$y_{ensemble}(x)=\sum_{k=\text{1}}^{k}w_{k}y_{k}(x),\;K=\text{4}$$ (1)

Here, $w_{k}$ denotes the weight assigned to the k-th base learner. $w_{k}$ are constrained such that $w_{k}\geq \text{0}$ and $\sum_{k=\text{1}}^{k}w_{k}=\text{1}$. The optimal weight vector w* is obtained by maximizing the validation performance of the ensemble:


$$w^{*}=\text{arg}maxR^{\text{2}}(y_{val}{,\hat{y}}_{ens,val}(w))$$ (2)

where w* represents the optimal ensemble weights, w represents any candidate weight combination, $y_{val}$ denotes the observed target values in the validation set, and ${\hat{y}}_{ens,val}(w)$ denotes the corresponding ensemble predictions generated by the weighted combination of the four base learners. The objective is to identify the weight vector that yields the highest validation $R^{\text{2}}$. During optimization, BOA iteratively evaluates candidate solutions, learns the dependency structure among high-performing candidates using a Bayesian network, and samples new candidate weight vectors until convergence is reached or the maximum number of iterations is met. This procedure enables efficient identification of the weight configuration that yields the best validation performance and improves the robustness of the ensemble model.

### 3.4. Shapley additive exPlanations (SHAP)

SHAP is a unified framework rooted in cooperative game theory. Initially proposed by Lundberg and Lee [32] (2017) as a model interpretation approach, it has subsequently been developed into a Python package and designed to explain predictions from complex ML models by accurately attributing feature contributions. Unlike conventional interpretation tools, SHAP provides mathematically consistent explanations by computing Shapley values—a concept originally formalized in economics to evaluate individual contributions within collaborative systems. It quantifies how each feature shifts a model’s baseline prediction toward the final output, ensuring both global interpretability (overall feature importance) and local transparency. This technique has been successfully applied in recent drought studies to identify key driving factors and interpret complex model behaviors. For instance, Feng et al. [33] (2025) utilized SHAP to quantify the impact of diurnal temperature range on short-term drought dynamics across China, while Xue et al. [34] applied the XGBoost-SHAP framework to identify drivers of forest drought sensitivity. SHAP’s model-agnostic property enables its application across diverse algorithms. This versatility significantly enhances the trustworthiness of black-box models. By balancing accuracy and interpretability, it bridges the gap between predictive performance and actionable insights.

### 3.5. Drought analysis techniques

#### 3.5.1. Standardized Precipitation Evapotranspiration Index (SPEI).

The SPEI enhances drought assessment by integrating temperature-driven PET with precipitation, addressing a critical limitation of the temperature-agnostic Standardized Precipitation Index (SPI). This dual-variable approach enables SPEI to reflect both moisture supply (precipitation) and atmospheric demand (PET), making it particularly sensitive to warming-induced aridification. Its multi-scalar design (1- to 48-month scales) supports versatile drought monitoring: shorter scales (e.g., SPEI-1) capture rapid meteorological droughts, while intermediate scales (e.g., SPEI-3 and SPEI-6) align with agricultural and ecological response timelines. The SPEI-3 was selected here to balance early drought detection and vegetation moisture stress dynamics. The SPEI drought classification is shown in Table 2.

**Table 2:** Drought classification criteria based on SPEI values.

Drought Grade	Drought Condition	SPEI
I	No drought	−0.5 < SPEI
II	Light drought	-1 ≤SPEI≤ -0.5
III	Moderate drought	-1.5 ≤SPEI≤ -1
IV	Severe drought	-2 ≤SPEI≤ -1.5
V	Extreme drought	SPEI <−2

The calculation steps of SPEI are as follows [6]:

Monthly PET was calculated using the Thornthwaite method [35]:


$$\text{PET}=\text{16K}{\left(\frac{\text{10T}}{\text{I}}\right)}^{\text{m}}$$ (3)

In Equation (3), K is the correction factor based on latitude, T is the monthly average temperature, I is the total heating index, and m is a constant.


$$\text{I}=\sum_{\text{i}=\text{1}}^{\text{12}}{\left(\frac{\text{T}}{\text{5}}\right)}^{\text{1}.\text{514}}$$ (4)

$$\text{m}=\text{6}.\text{75}\times {\text{10}}^{-\text{7}}{\text{I}}^{\text{3}}-\text{7}.\text{71}\times {\text{10}}^{-\text{5}}{\text{I}}^{\text{2}}+\text{1}.\text{792}\times {\text{10}}^{-\text{2}}\text{I}+\text{0}.\text{49}$$ (5)

Calculate the difference between precipitation (P) and PET for each month.


$${\text{D}}_{\text{i}}={\text{P}}_{\text{i}}-{\text{PET}}_{\text{i}}$$ (6)

Equation (6) defines ${\text{P}}_{\text{i}}$ and ${\text{PET}}_{\text{i}}$ as precipitation (P) and potential evapotranspiration (PET) at the monthly scale, respectively, with subscript i denoting the temporal index of the month. ${\text{D}}_{\text{i}}$ is water deficit series.

The SPEI is obtained by transforming the fitted water deficit series to a standard normal distribution:


$$\text{SPEI}=\text{w}-\frac{{\text{C}}_{\text{0}}+{\text{C}}_{\text{1}}\text{w}+{\text{C}}_{\text{2}}{\text{w}}^{\text{2}}}{\text{1}+{\text{d}}_{\text{1}}\text{w}+{\text{d}}_{\text{2}}{\text{w}}^{\text{2}}+{\text{d}}_{\text{3}}{\text{w}}^{\text{3}}},\text{w}=\sqrt{-\text{2ln}(\text{p})}$$ (7)

When precipitation (𝑃) ≤0.5, 𝑃 = 1 − 𝐹(𝑥). The 𝐹(𝑥) represents the probability distribution function of the D series. When 𝑃 > 0.5, P = 1 − P and the sign of the SPEI is reversed. The other constants in the formula are: ${\text{C}}_{\text{0}}=\;\text{2}.\text{515517}$, ${\text{C}}_{\text{1}}=\;\text{0}.\text{802853}$, ${\text{C}}_{\text{2}}=\;\text{0}.\text{010328}$, ${\text{d}}_{\text{1}}=\;\text{1}.\text{432788}$, ${\text{d}}_{\text{2}}=\;\text{0}.\text{189269}$ and ${\text{d}}_{\text{3}}=\text{0}.\text{001308}$.

Following widely used SPEI severity conventions [8,36], we converted continuous SPEI values into categorical drought severity classes using the threshold ranges listed in Table 2. Specifically, the model first predicts continuous SPEI at each timescale, and drought classes are then assigned by thresholding the predicted SPEI. For classification evaluation, observed drought classes were derived by applying the same thresholds to station-based SPEI, and the predicted classes were compared against these reference classes.

#### 3.5.2. Development of drought analysis indicators.

Drought prediction requires reconciling hydrological memory (soil moisture) with real-time biophysical stress signals (vegetation, evapotranspiration). This study employs Pa and PCI to quantify moisture deficits relative to climatological baselines. Pa standardizes precipitation deviations across monthly to seasonal scales, thus capturing abrupt meteorological droughts, while PCI isolates persistent precipitation anomalies that exceeding interannual variability. Complementing these indices, Vegetation Supply Water Index (VSWI) monitors canopy water supply status through EVI-LST coupling, where declining VSWI reflects stomatal regulation under soil moisture depletion—a precursor to agricultural drought. Simultaneously, SMCI tracks subsurface water storage anomalies, critical for identifying delayed hydrological droughts. ET links these realms, encoding the balance between atmospheric evaporative demand and root-zone water supply constraints.

The ML framework integrates these predictors by identifying their synergistic roles in drought detection. Response indicators such as Pa and VSWI capture immediate moisture deficits and vegetation stomatal behavior. In contrast, the PCI and SMCI describe prolonged hydrological imbalances. Vegetation indices (LAI, VCI, VHI) further delineate drought-induced physiological adaptations: VHI combines thermal and photosynthetic stress signals, whereas LAI quantifies canopy structural responses to water scarcity. This study also incorporates the TCI, PDSI, and PET. By accounting for nonlinear interactions, the framework avoids static variable weighting and instead adaptively maps drought patterns. The formulae and references for input parameter variable are given in Table 3.

**Table 3:** Definition, calculation formula, and source references of drought monitoring indices used in this study.

Drought indices	Formula	Ref
PCI	$\text{PCI}=\frac{{\text{P}}_{\text{i}}-{\text{P}}_{\text{min}}}{{\text{P}}_{\text{max}}-{\text{P}}_{\text{min}}}$	[3]
Pa	$\text{Pa}=\frac{{\text{P}}_{\text{i}}-\overset{―}{\text{P}}}{\overset{―}{\text{P}}}$	[36]
TCI	$\text{TCI}=\frac{{\text{LST}}_{\text{i}}-{\text{LST}}_{\text{min}}}{{\text{LST}}_{\text{max}}-{\text{LST}}_{\text{min}}}$	[37]
VCI	$\text{VCI}=\frac{{\text{NDVI}}_{\text{i}}-{\text{NDVI}}_{\text{min}}}{{\text{NDVI}}_{\text{max}}-{\text{NDVI}}_{\text{min}}}$	[38]
VHI	VHI=αVCI+(1−α)TCI (α denotes a constant value set to 0.5)	[38]
VSWI	$\text{VSWI}=\frac{\text{NDVI}}{\text{LST}}$	[39]
SMCI	$\text{SMCI}=\frac{{\text{SM}}_{\text{i}}-{\text{SM}}_{\text{min}}}{{\text{SM}}_{\text{max}}-{\text{SM}}_{\text{min}}}$	[40]

### 3.6. The process of building the model

**Fig 2:**
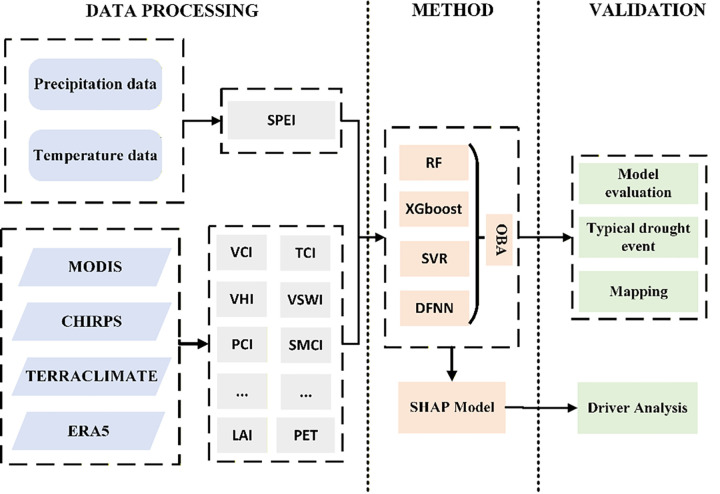
Flowchart of the drought prediction model construction.

The comprehensive drought monitoring model must consider the combined effects of moisture, temperature, vegetation, and other factors, as drought is influenced by multiple interacting variables. To leverage the advantages of multi-source data and multi-model integration, we selected over 10 variables as predictors, including PCI, Pa, TCI, VCI, VHI, VSWI, SMCI, LAI, ET and other related variables.. To ensure stable model performance, all input features were normalized to a [0, 1] range using Min-Max scaling prior to model training. To address potential multicollinearity, we calculated the Pearson correlation coefficients among all predictor pairs (Fig 7). We excluded drivers with excessively strong correlations. The dataset was randomly divided into a training set (75%) and a testing set (25%). The spatiotemporal matching of data, calculation of driving factors, normalization, and training-set partitioning are all abstracted as the “DATA PROCESSING” step in the Flowchart (Fig 2). To ensure the reliability of the results, cross-validation was used for model validation and hyperparameter tuning. Four machine learning models (RF, XGBoost, SVR, and DFNN) were trained on the training set, and their optimal hyperparameters were selected through grid search. BOA was employed to determine the optimal weighting for the ensemble model, which was then used to generate final predictions. Model performance (for both individual models and the ensemble) was evaluated on the testing set using the coefficient of determination (R^2^), root mean square error (RMSE), and mean absolute error (MAE). Correlation analysis and SHAP were employed to analyze variable relationships and feature importance. Although both correlation analysis and SHAP relate predictors to drought responses, they answer different questions and therefore complement each other. In addition, predictive uncertainty was proxied by the standard deviation (std) of the predictions from the four base learners (RF, XGBoost, SVR, and DFNN), where larger std values indicate greater disagreement among models and thus higher uncertainty. Finally, spatiotemporal drought distribution maps were generated by integrating historical drought years and critical drought periods, enabling spatialtemporal analysis of drought patterns. The detailed model construction process is illustrated in Fig 2.

### 3.7. Model evaluation

The drought prediction performance is evaluated by comparing simulated and observed drought indices. Three commonly used performance metrics are calculated including$R^{\text{2}}$, RMSE, MAE:


$${\text{R}}^{\text{2}}=\frac{{\left(\sum \left(\text{p}-\overset{―}{\text{p}}\right)(\text{m}-\overset{―}{\text{m}})\right)}^{\text{2}}}{\sum {\left(\text{p}-\overset{―}{\text{p}}\right)}^{\text{2}}\sum {\left(\text{m}-\overset{―}{\text{m}}\right)}^{\text{2}}}$$ (6)

$$\text{RMSE}=\sqrt{\frac{\sum (\text{m}-\text{p}{)}^{\text{2}}}{\text{n}}}$$ (7)

$$\text{MAE}=\frac{\sum \left|\text{p}-\text{m}\right|}{\text{n}}$$ (8)

where m and p refer to the measured and predicted values, respectively, and the overbar denotes the mean value.

## 4. Results

### 4.1. Spatial and temporal evolution characteristics of precipitation

**Fig 3:**
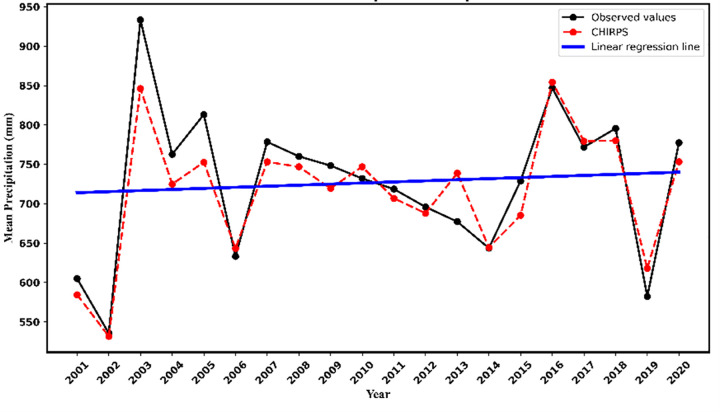
A comparison of annual total rainfall (mm) recorded by 30 ground-based observation stations with CHIRPS data, along with their linear regression curves, is presented.

**Fig 4:**
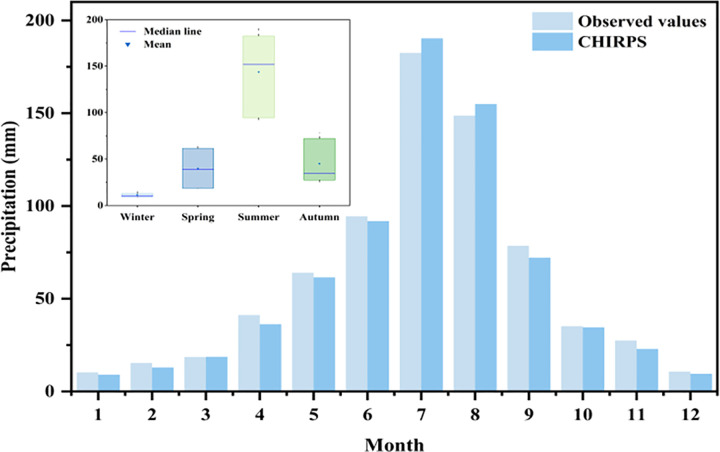
A comparison of the monthly mean rainfall (mm) data with CHIRPS records. The inset in the upper left corner shows boxplots of precipitation in spring, summer, autumn, and winter.

The spatiotemporal evolution of precipitation in the Beijing-Tianjin-Hebei-Shandong-Henan region from 2000 to 2020 was analyzed using station observations and CHIRPS data. Over the 20-year period, CHIRPS data exhibited consistent trends with station observations (Fig 3), indicating relatively stable interannual precipitation variations. The Mann-Kendall (M-K) test detected no significant abrupt changes or trends. Station data revealed a 20-year mean precipitation of 723.0 mm, with the highest variability between 2002 (annual minimum: 535.5 mm) and 2003 (annual maximum: 993.4 mm), yielding a ratio of 1.86. The absolute mean error between CHIRPS and station data was 26.0 mm (range: 3.8–87.2 mm). Precipitation displayed clear seasonal variations (Fig 4): summer rainfall peaked (monthly mean: 141.9 mm), particularly in July (182.5 mm) and August, while winter recorded the lowest values (monthly mean: 10.5 mm), especially in January (10.2 mm) and December. Seasonal rankings were: summer > autumn > spring > winter. Both station observations and CHIRPS data consistently captured these characteristics, demonstrating strong consistency in reflecting seasonal precipitation dynamics (RMSE = 34.07 mm, bias = −12.12 mm, R^2^ = 0.86).

**Fig 5:**
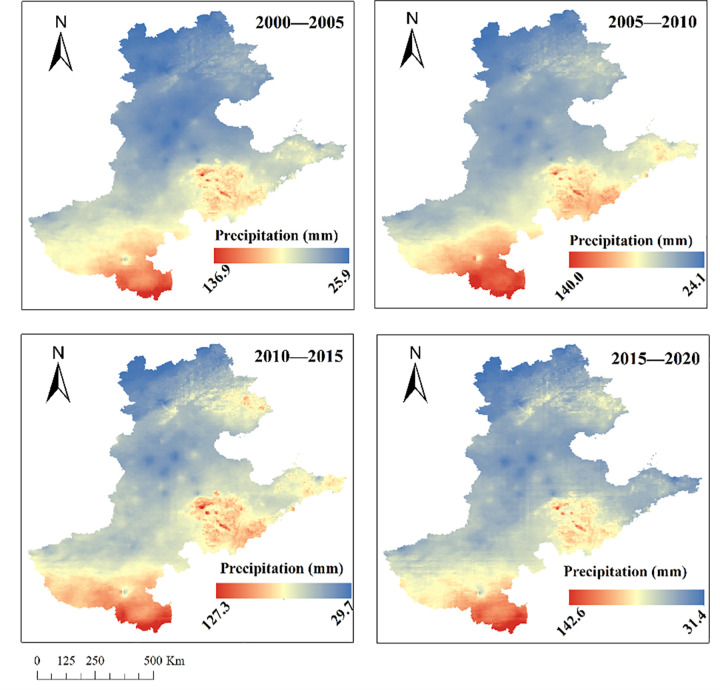
Maps illustrating the spatial distribution patterns of 5-year average rainfall in the Beijing-Tianjin-Hebei-Shandong-Henan Region from 2000 to 2020.

Over the past two decades, the spatial distribution pattern of precipitation in the Beijing-Tianjin-Hebei-Shandong-Henan region remained relatively stable (Fig 5). Specifically, rainfall exhibited a distinct south-north gradient, with higher precipitation concentrated in southern Henan Province—particularly in Xinyang, Zhumadian, and Nanyang cities—followed by southern and eastern Shandong. In contrast, Hebei Province, Beijing, and Tianjin generally experienced lower rainfall. Annual precipitation ranged between approximately 136.7 mm (maximum) and 27.8 mm (minimum), showing minimal interannual fluctuations. Notably, compared to the 2000–2005 period, northern areas, particularly the eastern regions adjacent to Beijing and Tianjin, had experienced rainfall reductions of varying degrees. Nevertheless, the overall spatial distribution pattern showed no significant changes.

### 4.2. Comparison of model performance of ML algorithms

**Fig 6:**
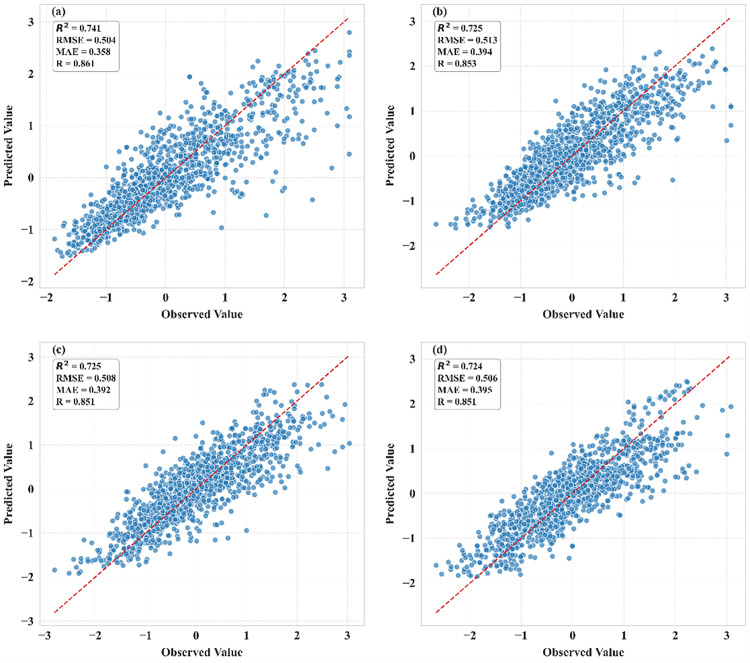
Scatter plots comparing drought predictions from an ensemble ML model with observed values across four spatial/temporal scales: **(a)** SPEI-1, **(b)** SPEI-3, **(c)** SPEI-6, and **(d)** SPEI-12, respectively.

To evaluate the drought prediction capabilities of different models in the Beijing-Tianjin-Hebei-Shandong-Henan region, performance in forecasting SPEI indices across four temporal scales (SPEI-1, SPEI-3, SPEI-6, SPEI-12) was compared. Results demonstrated that the ensemble model optimized through Bayesian algorithm outperformed individual ML models, achieving superior metrics across all scales (Fig 6). The ensemble model yielded R^2^ values of 0.743 (SPEI-1), 0.725 (SPEI-3), 0.714 (SPEI-6), and 0.719 (SPEI-12), with SPEI-1 showing the best predictive performance (RMSE: 0.502; MAE: 0.361), while SPEI-6 exhibited relatively weaker results (RMSE: 0.518; MAE: 0.405). The correlation coefficients for all four scales remained above 0.850. Among individual models, RF performs optimally in most scenarios, with the highest R^2^ for SPEI-1 (0.731), and the lowest RMSE (0.513) and MAE (0.368), with statistically significant differences compared to other models. It also maintains an R^2^ advantage for SPEI-12 (0.710). DNN demonstrates competitiveness in mid-term predictions, with the R^2^ for SPEI-3 (0.717) slightly surpassing RF (0.717), and the RMSE (0.520) and MAE (0.398) for SPEI-6 being the lowest among all models. SVR shows an R^2^ for SPEI-1 (0.731) close to that of RF and the lowest MAE (0.366). XGBoost generally performs weakly, with the lowest R^2^ for SPEI-1 (0.721) and SPEI-12 (0.6883) among the models. We also compared the proposed framework with two additional baselines (Linear Regression and LSTM; S2 Table). Linear Regression showed moderate performance (R^2^ = 0.525–0.661; RMSE = 0.602–0.678), whereas LSTM performed noticeably worse and less stably across scales (R^2^ = 0.073–0.459; RMSE = 0.724–1.071). Overall, the BOA-optimized ensemble remained consistently superior across all four SPEI timescales. The R^2^ and RMSE values mentioned above are the averages across all stations and for the period from 2001 to 2020, based on five-fold cross-validation. All models show a downward trend in R^2^ as the scale increases (e.g., RF decreases by 0.021), indicating an increase in the complexity of long-term drought prediction. In conclusion, RF is a robust choice for multi-scale SPEI prediction (P < 0.05), while DNN shows application potential at specific scales.

We further quantified predictive uncertainty, and the uncertainty patterns across the four SPEI timescales are shown in S3 Fig. The mean uncertainty values were 0.113 for SPEI-1, 0.111 for SPEI-3, 0.119 for SPEI-6, and 0.133 for SPEI-12. These results indicate that predictive uncertainty was lowest for SPEI-3 and highest for SPEI-12, suggesting that model agreement decreased as the drought timescale increased. For operational drought monitoring, these uncertainty estimates can be used as a confidence indicator, with lower values reflecting stronger agreement among base learners and higher values requiring more cautious interpretation of the prediction results.

#### 4.2.1. Drought consistency analysis.

According to the drought classification criteria in Table 2, the consistency between drought severity levels predicted by the ensemble model and observed values was statistically analyzed across 30 stations in the Beijing-Tianjin-Hebei-Shandong-Henan region from 2000 to 2020, with a total of 5016 samples (Table 4). The ensemble ML model demonstrated superior performance in drought severity prediction, achieving consistency rates above 78% across all four temporal scales. Extreme drought scenarios showed the highest agreement rate of over 98%, while mild drought predictions exhibited the lowest consistency at approximately 80%. The exceptionally high (>98%) agreement for extreme drought may indicate overfitting due to its limited samples. For SPEI-3 and SPEI-6 scales, the agreement rates were 78% and 79%, respectively. Among all scales, SPEI-1 achieved the highest classification consistency rate, whereas SPEI-6 showed the lowest. Nevertheless, the model maintained robust consistency in drought severity classification across all evaluated temporal resolutions. We further tested the sensitivity of drought classification to threshold choices by perturbing the SPEI cutoffs in Table 2 by ±0.1 and ±0.2 and recomputing the drought classes and classification metrics. The main conclusions remained stable across these perturbations.

**Table 4:** Drought categorization consistency rate at each scale (n = 5016).

Consistency Rate	SPEI-1	SPEI-3	SPEI-6	SPEI-12
No drought	0.88	0.84	0.85	0.86
Light drought	0.81	0.78	0.79	0.83
Moderate drought	0.91	0.89	0.89	0.88
Severe drought	0.97	0.97	0.96	0.95
Extreme drought	0.98	0.99	0.98	0.98

#### 4.2.2. Correlation Analysis Based on Input Variables.

**Fig 7:**
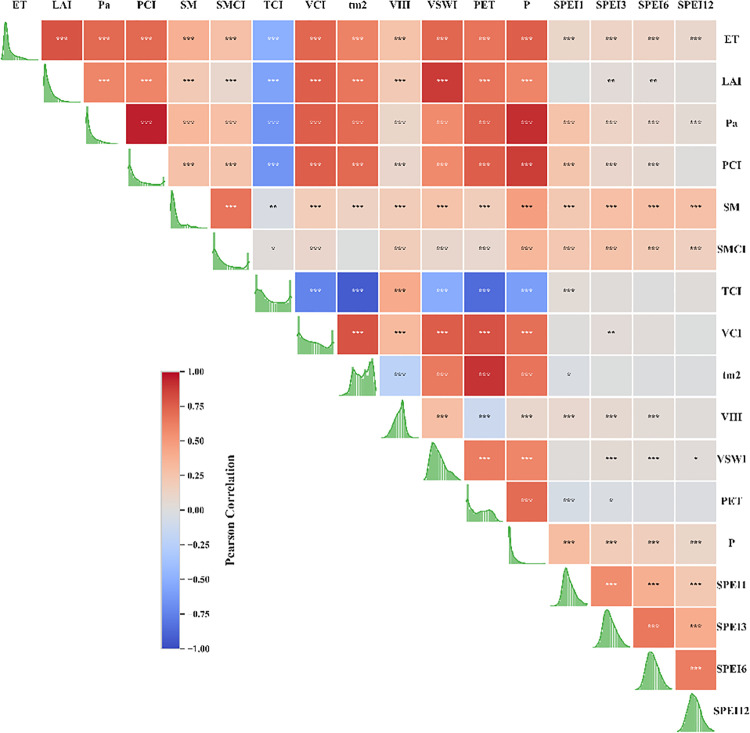
Correlation heatmap (* indicates significance, ***, **, * represent P < 0.001, P < 0.01, P < 0.05 respectively), with histograms of frequency distributions for each variable displayed along the diagonal.

Drought is influenced by various factors, including precipitation, soil environment, and vegetation, which affect drought at different scales. Therefore, we calculated multiple remote sensing indices as evaluation factors. Correlation coefficients (R) between various factors and the SPEI index, as well as correlations among the factors, were calculated, and frequency distribution plots for each variable were generated to systematically evaluate the necessity of multi-source data integration in drought monitoring (Fig 7). Precipitation exhibited the highest correlation with SPEI, although the relationship was relatively weak (R = 0.31, P < 0.001), and its influence diminished with increasing temporal scales. Similarly, PCI and Pa showed higher correlations with short-term drought (SPEI-1) at R = 0.27 and R = 0.25, respectively (P < 0.001). Soil moisture (SM) was the most important factor for SPEI-6 (R = 0.30, P < 0.001), with correlations of 0.29 and 0.27 with SPEI-3 and SPEI-12, respectively, indicating its heightened relevance for monitoring agricultural drought over prolonged periods. Frequency distributions revealed distinct characteristics: LAI, SM, and precipitation displayed higher frequencies at lower values, reflecting generally low rainfall, soil moisture, and vegetation leaf area across the Beijing-Tianjin-Hebei-Shandong-Henan region. In contrast, drought indices approximated a normal distribution.

### 4.3. Drought driver analysis based on SHAP

**Fig 8:**
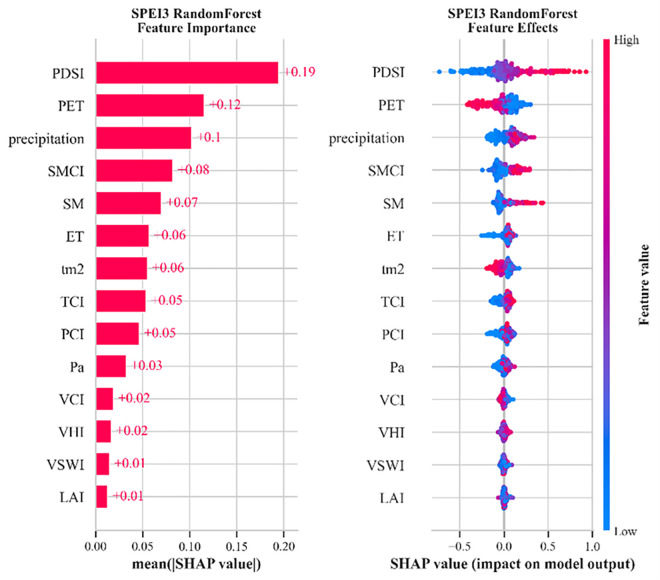
Bar and hive plots depicting variable importance based on SHAP values for the RF model’s prediction of SPEI-3 (SPEI-3 characterizes agricultural drought).

Although correlation analysis summarizes pairwise linear associations between individual predictors and SPEI, SHAP complements it by providing model-based, conditional feature attributions that account for nonlinear effects and interactions. To analyze the driving effects of different factors on drought, we conducted explainable ML using the SHAP library on the top-performing RF model. For short-term drought (SPEI-1), PET and precipitation contributed most significantly at 21% each, followed by temperature and SM (S4 Fig). In long-term drought assessment (timescales ≥3 months), PDSI emerged as the most critical factor, with contribution rates of 19% for SPEI-3 (Fig 8), 29% for SPEI-6 (S5 Fig), and 39% for SPEI-12 (S6 Fig). S4–S6 Figs are provided in the supplementary file. This highlights the importance of reanalysis-based drought indicators in drought monitoring. However, because PDSI is strongly collinear with SPEI-related information, its contribution was not emphasized in the physical interpretation. Instead, PET, precipitation, and soil moisture (SM) were discussed as the main interpretable drivers of long-term drought, in descending order of contribution and consistent with the correlation analysis. Vegetation indices (LAI, VCI), VHI, and VSWI exhibited the lowest contributions, potentially due to anthropogenic interventions in agricultural landscapes dominating the Beijing-Tianjin-Hebei-Shandong-Henan region, which may obscure vegetation-related drought signals.

From a physical-process and drought-severity perspective, the identified SHAP drivers are consistent with expected drought mechanisms across timescales. Shorter-timescale drought (e.g., SPEI-1/3) is mainly controlled by rapid meteorological forcing, including precipitation-related water supply variability and atmospheric evaporative demand, whereas longer-timescale drought (e.g., SPEI-6/12) increasingly reflects land-surface memory and accumulated deficits captured by soil-moisture-related indicators. With increasing drought severity, persistence- and demand-related factors are expected to play a stronger role, while mild drought conditions tend to be more sensitive to short-term fluctuations in meteorological forcing (S4 and S6 Fig).

### 4.4. Spatial variability of drought

**Fig 9:**
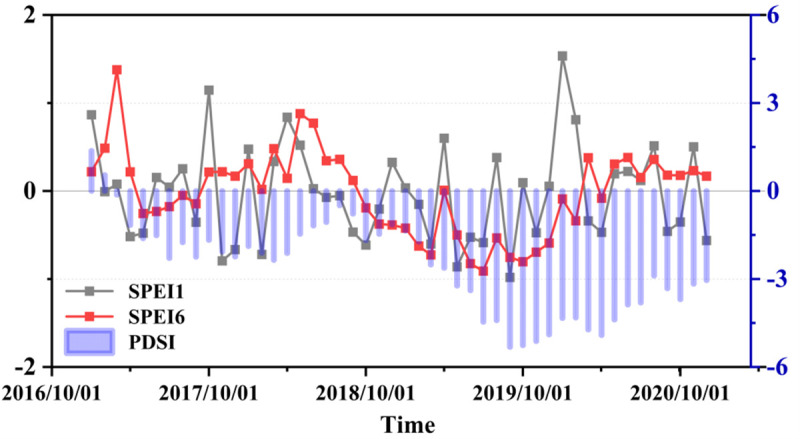
Time series curves of two temporal scales (SPEI-1, SPEI-6) and sites PDSI bar charts calculated from observational data (2017–2020).

This study developed a drought monitoring model using ensemble ML to assess and classify drought conditions in the Beijing-Tianjin-Hebei-Shandong-Henan region. Additionally, we focused on analysing the spatiotemporal variations during the summer maize growing season from 2017 to 2020, as shown in Fig 9. By integrating station-observed data to calculate monthly drought index averages, comparative analysis of SPEI and PDSI revealed heightened drought severity in 2019 (Fig 9). Drought-prone months primarily clustered around October, while spring and early summer exhibited relatively higher moisture levels. Given the vast croplands in the Beijing-Tianjin-Hebei-Shandong-Henan region, where maize cultivation prevails with sowing in June and harvesting in September, our analysis concentrated on the spatiotemporal drought dynamics during the 2019 maize-growing season (June-September).

The optimized ensemble model was applied to predict drought conditions across the Beijing-Tianjin-Hebei-Shandong-Henan region, classifying drought severity into five levels based on SPEI criteria in Table 2: Extreme drought, Severe Drought, Moderate Drought, Light Drought, and No Drought. Two representative temporal scales, short-term (SPEI-1) and medium-to-long-term (SPEI-6), were selected for monitoring and analysis.

**Fig 10:**
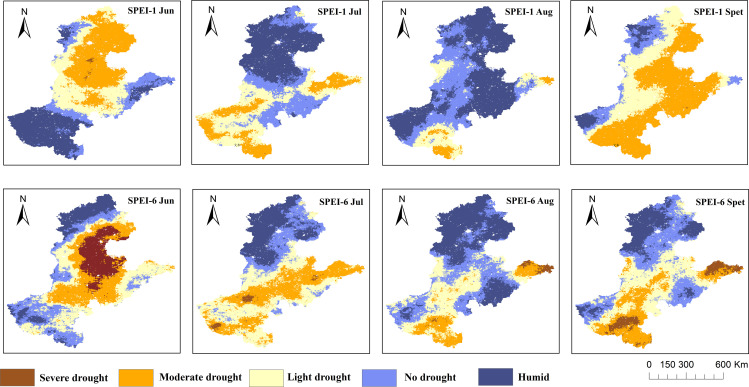
Spatial Distribution of Drought Severity Classifications at Two Temporal Scales (SPEI-1 and SPEI-6) in the Beijing-Tianjin-Hebei-Shandong-Henan Region.

The spatiotemporal distribution of drought in the study area in 2019 is shown in Fig 10. In the four analyzed months (Jun, Jul, Aug, and Sept), three months, excluding Aug with relatively mild drought, experienced widespread drought. In June 2019, the model identified severe drought concentrated in the northern part of the region, a finding that is empirically supported by a report from the Chinese Central People’s Government (https://www.gov.cn/xinwen/2019-07/05/content_5406452.htm), which stated that 11.9 million acres of crops in Hebei suffered from drought in 2019, with the reported affected area closely aligning with our model’s output. The modeled drought patterns show strong agreement with documented historical events, thereby validating the model’s effectiveness. By July, drought conditions eased in northern areas but intensified as moderate drought in eastern Shandong and Henan. In September, drought further expanded: SPEI1 identified extensive moderate drought in eastern Beijing-Tianjin-Hebei-Shandong-Henan, while SPEI6 detected severe drought in Weihai and Yantai (Shandong Province) and significant drought severity in southern Henan. This methodology offers a robust framework for drought mitigation and analysis in the Beijing-Tianjin-Hebei-Shandong-Henan region.

## 5. Discussion

Data-driven models have proven effective for drought monitoring [41]. Recent ensemble ML applications, such as the ensemble deep RVFI model [42] and Multi-Model Ensemble [43], have demonstrated robust drought prediction performance. This study explores ensemble machine learning for drought analysis, achieving higher classification accuracy and addressing model performance variability. Our ensemble model’s performance, while slightly lower than that of the most advanced ensemble deep RVFI model (R > 0.9) and the hybrid deep learning framework [44], offers a favorable trade-off through its significantly simpler architecture and reduced computational demands. It is noteworthy that the prediction task in our study is inherently more challenging. Additionally, SHAP addressed ML’s “black-box” limitation by quantifying driver importance [45], while ML retains advantages in parameter simplicity and computational efficiency over complex deep learning [46]. However, limitations persist in capturing intricate interactions between meteorological indices and remote sensing variables [47]. This is also likely one of the reasons why the model’s performance decreases as the drought timescale lengthens.

From 2000 to 2020, despite stable precipitation trends, rising temperatures enhanced evapotranspiration, amplifying drought risks, consistent with the model’s identification of precipitation and PET as dominant drivers. Physically, precipitation directly sustains soil water availability, while PET (fueled by warming) accelerates water loss from vegetation and soil, explaining why stable rainfall can still heighten drought vulnerability under rising temperatures. This threatens agricultural productivity in the Beijing-Tianjin-Hebei-Shandong-Henan region. Notably, mid- to long-term droughts (e.g., SPEI-6) were more severe than short-term events during June–September 2019 (Fig 10), with persistent intensification in southern Henan and eastern Shandong. This likely stems from climate change-induced shifts in rainfall frequency; short-term rainfall alleviates surface dryness but fails to reverse deep soil desiccation amid elevated PET. Because prolonged droughts directly restrict crop water uptake, a key mechanism underlying agricultural drought impacts, these results highlight the urgent need for targeted mitigation strategies to protect crop yields.

This study is a single-region case study, and performance may vary across climatic regimes and observation networks. Nevertheless, the framework is expected to be transferable because it relies on widely available hydroclimatic and remote-sensing predictors and uses a model-agnostic ensemble strategy; in a new region it can be re-calibrated with local station-based SPEI and minor predictor adjustments to reflect regional processes. In data-sparse areas, gridded reanalysis and satellite-only proxies can be used to construct a weak reference drought signal for initial deployment, with subsequent refinement once limited local labels become available. Multi-region evaluation will be pursued in future work to quantify generalization under domain shift.

Several limitations warrant consideration: (1) Coarse-resolution and temporally inconsistent input data (monthly scale) introduced estimation biases and constrained model performance, as evidenced by low correlations (R < 0.4) between predictors and drought indices. A key issue was the potential mismatch in temporal resolution: the monthly aggregation of data may fail to capture critical sub-monthly drought triggers and responses, particularly for short-term droughts, thereby smoothing out dynamics essential for accurate monitoring. (2) Furthermore, uncertainties inherent in the original remote sensing and meteorological datasets are likely propagated through our analytical chain, potentially amplifying the final uncertainty in drought severity classification. (3) Factors such as elevation and vegetation type, though influential in drought dynamics, were excluded due to the region’s predominant flat topography and cropland cover. Future studies should incorporate higher-resolution datasets and these omitted variables to refine drought monitoring frameworks.

## 6. Conclusion

A Bayesian-optimized ensemble ML framework integrating multi-source meteorological and biophysical data (precipitation, vegetation indices, soil moisture) is proposed for multi-scale drought monitoring in the intensive agricultural region of Beijing-Tianjin-Hebei-Shandong-Henan, China. Core findings from this study are summarized as follows: (1) The developed ensemble model delivers robust predictive performance for the SPEI across 1-, 3-, 6-, and 12-month scales, with R^2^ values ranging from 0.71 to 0.74, which outperform single-algorithm models by capturing complex nonlinear drought-driver relationships. (2) SHAP analysis enhances model interpretability by identifying scale-dependent dominant drivers: Pa (R = 0.31) and PET each contribute 21% to short-term drought (SPEI-1), while the PDSI emerges as the top predictor for long-term drought (39% contribution to SPEI-12), with soil moisture also exerting a significant influence on extended dry conditions. (3) Spatiotemporal characterization via the framework identifies severe drought clusters in the Beijing-Tianjin-Hebei region during the 2019 maize growing season, with SPEI-6 effectively capturing the persistent fan-shaped aridity zone west of the Bohai Sea. The model achieves over 78% accuracy in classifying drought intensities and 98% accuracy in extreme drought detection, demonstrating high reliability in practical applications.

This study contributes to drought monitoring methodology by integrating meteorological memory effects and real-time biophysical signals, providing a scalable and interpretable framework for intensive agricultural systems. The framework, with its fine spatiotemporal resolution and demonstrated predictive capability, offers a practical reference for regional drought assessment and presents a potential foundation for integration into early-warning systems, supporting data-driven proactive drought risk management.

## Supporting information

S1 Table Grid search for the best parameters. (DOCX)

S2 Table Performance comparison of two baselines (Linear Regression and LSTM) for multi-timescale SPEI prediction (R^2^ and RMSE). (DOCX)

S3 Fig Predictive uncertainty of multi-timescale SPEI predictions, std denotes the standard deviation. (a) SPEI-1, (b) SPEI-3, (c) SPEI-6, and (d) SPEI-12, respectively.
(PNG)

S4 Fig Bar and hive plots depicting variable importance based on SHAP values for the RF model’s prediction of SPEI-1. (PNG)

S5 Fig Bar and hive plots depicting variable importance based on SHAP values for the RF model’s prediction of SPEI-6. (PNG)

S6 Fig Bar and hive plots depicting variable importance based on SHAP values for the RF model’s prediction of SPEI-12. (PNG)
