# Supplementary material for: Integrated drought monitoring and analysis: A novel framework based on multi-source remote sensing data and ensemble machine learning
Source: PLoS One. 2026 Apr 21;21(4):e0346060. doi: 10.1371/journal.pone.0346060 (PMC13098985; doi:10.1371/journal.pone.0346060)
Supplement: S1 Table — (DOCX) [file pone.0346060.s001.docx]

**S1 Table. Grid search for the best parameters.**

| ML Algorithms | Hyperparameter ranges | Best Parameters |
| --- | --- | --- |
| Random Forest | n_estimators': [100, 300, 500, 800,1000], 'max_features': [0.2, 0.3, 'sqrt'], 'min_samples_split': [2, 5, 8], 'min_samples_leaf': [1, 2, 4], 'bootstrap': [False] | n_estimators=800, min_samples_split=8, min_samples_leaf=1, max_features=0.3, bootstrap=False, n_jobs=-1 |
| XGBoost | 'n_estimators': [100, 300, 500, 800,1000], 'learning_rate': [0.01, 0.03, 0.05], 'max_depth': [4, 6, 8], 'subsample': [0.6, 0.8, 1.0], 'colsample_bytree': [0.6, 0.8, 1.0] | n_estimators=800, learning_rate=0.03, max_depth=6, subsample=0.8, colsample_bytree=0.6, n_jobs=-1 |
| SVR | 'kernel': ['rbf'], 'C': [0.1, 1.0, 10, 100], 'gamma': ['scale', 0.1, 1], 'epsilon': [0.01, 0.1, 0.2] | kernel='rbf', C=1.0, epsilon=0.1 |
| DNN | 'batch_size': [32, 64, 128], 'learning_rate': [0.001, 0.01, 0.1], 'architecture': [(120-64-1), (120-32-10-1), (120-10-1), (64-32-10-1)], 'activation': ['relu', 'sigmoid'] | architecture: 120-10-1, activation: sigmoid, epochs=400, batch_size=64, validation_split=0.1, early_stopping patience=10 |
