# Supplementary material for: Integrated drought monitoring and analysis: A novel framework based on multi-source remote sensing data and ensemble machine learning
Source: PLoS One. 2026 Apr 21;21(4):e0346060. doi: 10.1371/journal.pone.0346060 (PMC13098985; doi:10.1371/journal.pone.0346060)
Supplement: S2 Table — (DOCX) [file pone.0346060.s002.docx]

**S2 Table. Performance comparison of two baselines (Linear Regression and LSTM) for multi-timescale SPEI prediction (R² and RMSE).**

| SPEI | LinearReg_R^2^ | LinearReg_RMSE | LSTM_R^2^ | LSTM_RMSE |
| --- | --- | --- | --- | --- |
| SPEI1 | 0.6613 | 0.6018 | 0.0732 | 1.0713 |
| SPEI3 | 0.6095 | 0.635 | 0.3535 | 0.8171 |
| SPEI6 | 0.5474 | 0.6646 | 0.3432 | 0.8006 |
| SPEI12 | 0.5253 | 0.6779 | 0.4585 | 0.7241 |
