# Supplementary figures and images for: Integrated drought monitoring and analysis: A novel framework based on multi-source remote sensing data and ensemble machine learning
Source: PLoS One. 2026 Apr 21;21(4):e0346060. doi: 10.1371/journal.pone.0346060 (PMC13098985; doi:10.1371/journal.pone.0346060)

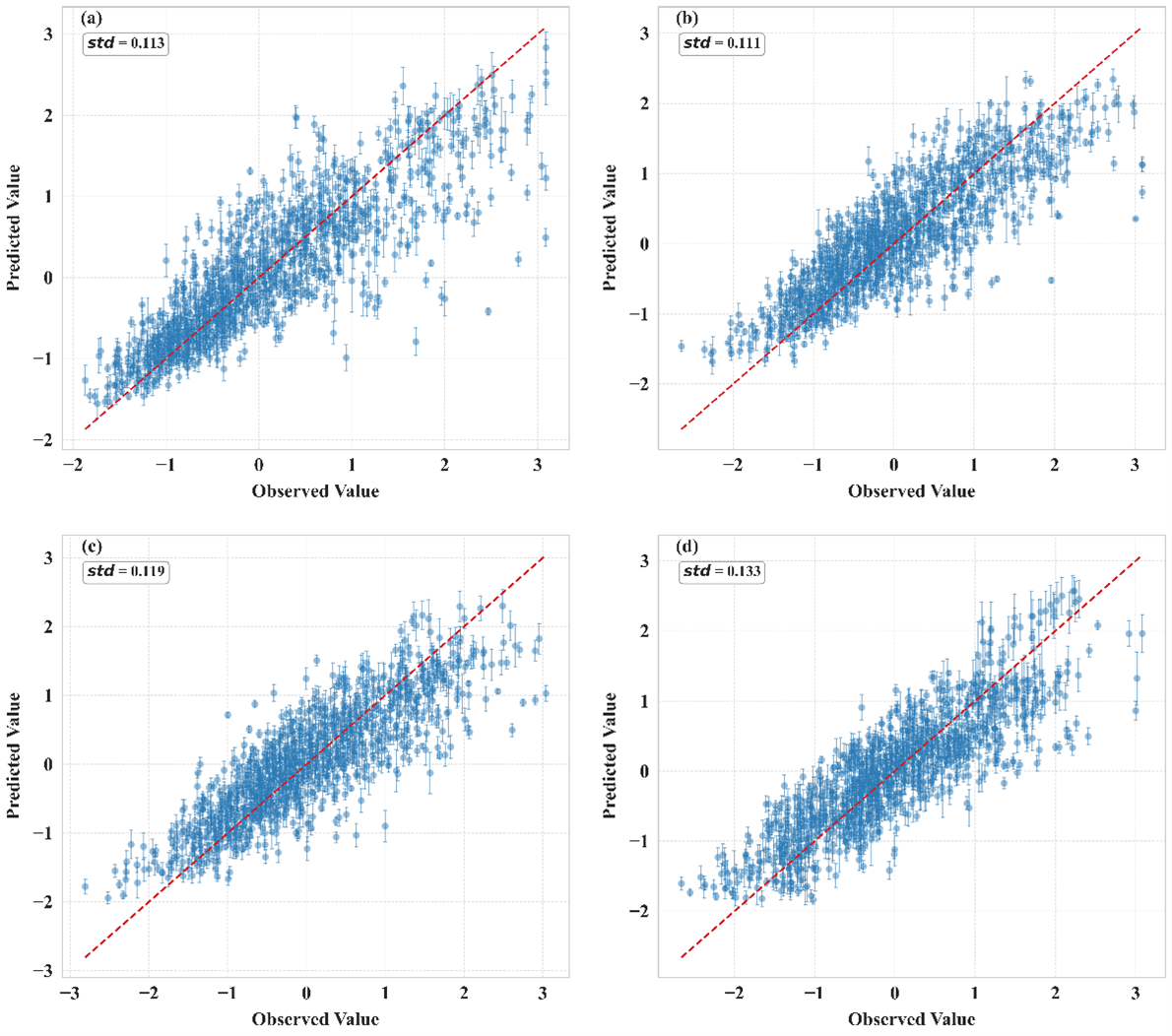

Supplement: S3 Fig — (a) SPEI-1, (b) SPEI-3, (c) SPEI-6, and (d) SPEI-12, respectively. (PNG) [file pone.0346060.s003.png]

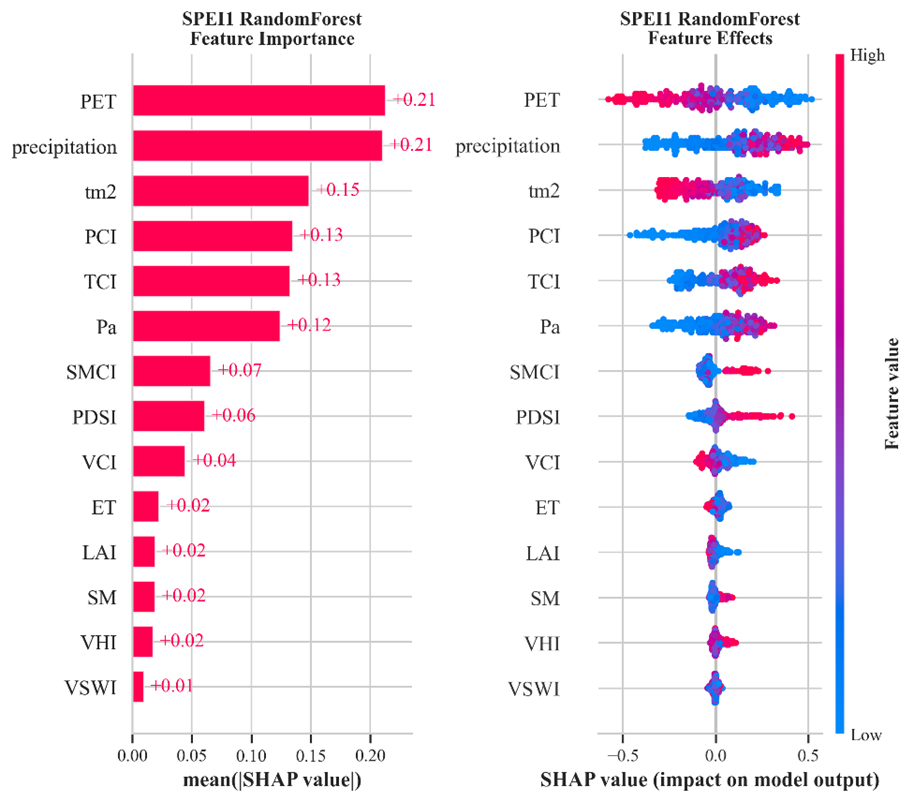

Supplement: S4 Fig — (PNG) [file pone.0346060.s004.png]

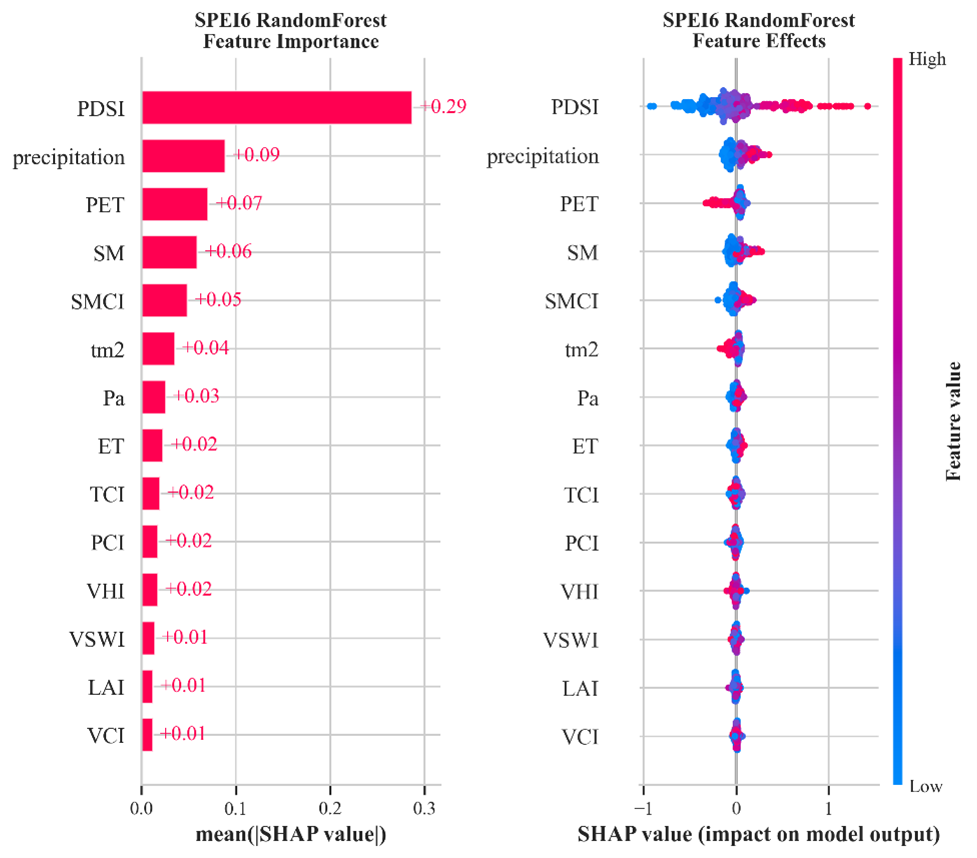

Supplement: S5 Fig — (PNG) [file pone.0346060.s005.png]

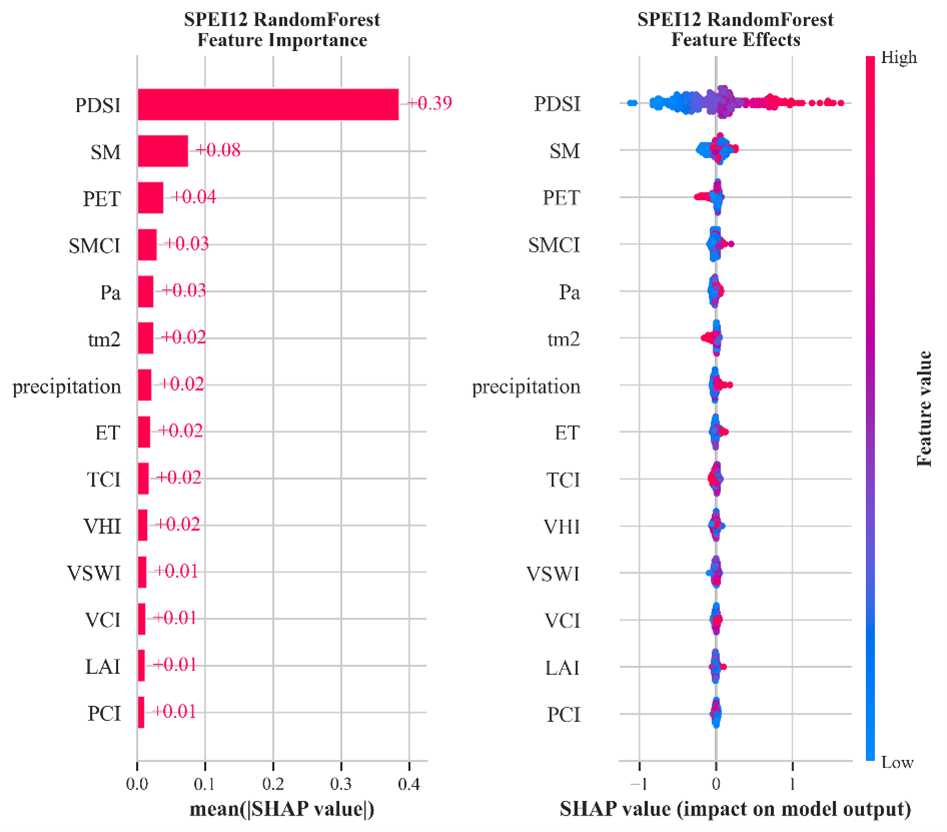

Supplement: S6 Fig — (PNG) [file pone.0346060.s006.png]
